# Listening Without the Noise: Near‐Silent Looping Star fMRI Reveals Neural Processing of Degraded Speech

**DOI:** 10.1002/hbm.70501

**Published:** 2026-03-12

**Authors:** Christopher J. Ritter, Alejandra M. Hüsser, András Jakab, Florian Wiesinger, Ana Beatriz Solana, Brice Fernandez, Huw Swanborough, Ruth O’Gorman Tuura, Alexis Hervais‐Adelman

**Affiliations:** ^1^ Department of Basic Neurosciences University of Geneva Geneva Switzerland; ^2^ Zurich Center for Linguistics University of Zurich Zurich Switzerland; ^3^ Center for MR‐Research University Children’s Hospital Zurich Zurich Switzerland; ^4^ Department of Neuroimaging King’s College London London UK; ^5^ GE HealthCare Munich Germany; ^6^ GE HealthCare Buc France

**Keywords:** degraded speech, fMRI, looping star, near‐silent

## Abstract

Excessive acoustic noise during fMRI poses challenges for auditory cognitive neuroscience. We aimed to evaluate a near‐silent fMRI sequence, Looping Star (LS), for measuring brain responses to acoustically degraded speech. We hypothesized that LS would maintain BOLD sensitivity comparable to conventional Gradient‐echo EPI and potentially reveal enhanced activation in brain regions associated with effortful speech processing under degraded listening conditions. Ten healthy adult native German speakers underwent fMRI while passively listening to blocks of spoken sentences under four conditions: clear speech, moderately degraded speech (spectral noise vocoding at ~50% intelligibility), unintelligibly degraded speech and clear speech mixed with EPI‐like scanner acoustic noise. Each participant was scanned in four runs (two using standard single‐echo EPI and two using the near‐silent multi‐echo LS sequence) in an alternating order. All fMRI timeseries (single‐echo EPI, single‐echo LS and echo‐combined LS) detected robust bilateral auditory cortex activation for all Sound > silent baseline. Echo‐combined LS, however, yielded larger activation clusters and higher peak effect sizes, extending beyond auditory cortex regions. Notably, only echo‐combined LS revealed significant activation in the left inferior frontal gyrus (inferior frontal gyrus, pars opercularis) and adjacent regions when comparing all speech stimuli to baseline, whereas EPI and single‐echo LS showed no such frontal activation. Direct between‐sequence comparisons demonstrated significantly greater BOLD responses in echo‐combined LS compared to EPI in key regions (including left insula, left precentral gyrus and left inferior frontal gyrus) for the all Sound > baseline and moderately degraded > baseline contrasts. These findings underscore the promise of near‐silent fMRI for auditory neuroscience: by virtually eliminating scanner noise, LS fMRI can reveal neural responses to degraded speech that might be masked during loud EPI scans.

## Graphical abstract

The near‐silent Looping Star fMRI sequence is validated and directly compared to standard EPI in a degraded speech paradigm. Combined‐echo LS yielded larger BOLD activation clusters which extended into key regions associated with effortful processing of degraded speech (insula, precentral gyrus, IFG), while standard EPI and single‐echo LS did not.

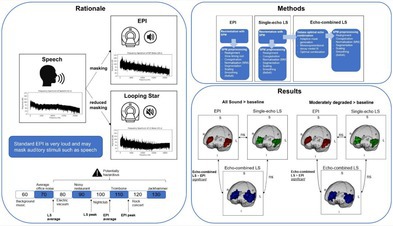

## 1 Introduction

Gradient‐echo echo‐planar imaging (EPI), the most commonly used fMRI sequence, produces intense acoustic noise on the order of 100 dB (peaking above 110 dB at 3 T) (Damestani et al. 2021; Hattori et al. 2007; Moelker and Pattynama 2003). This noise results from vibrations induced by rapidly switching magnetic field gradients (via eddy currents and Lorentz forces on the scanner's coils) (Mansfield et al. 1998; Moelker and Pattynama 2003; Mollasadeghi et al. 2013) and despite efforts in hardware design, acoustic padding and sequence optimization, substantial background noise remains an inherent by‐product of standard EPI scans (AlMeer 2022; McJury 2022; Radomskij et al. 2002). Such noise not only poses a risk to hearing (necessitating uncomfortable ear protection (Price et al. 2001)), but is also generally unpleasant for participants (Skouras et al. 2013).

In the brain, speech comprehension under ideal circumstances is both effortless and does not need attentional processes (Crinion et al. 2003; Wild et al. 2012). Processing of clearly intelligible speech has been shown to recruit lateral regions of the temporal cortex, including the bilateral superior temporal gyri and the left middle and inferior temporal gyri (Saur et al. 2008). Yet, speech presented under controlled conditions does not reflect everyday listening, where speech signals are seldom perceived in the absence of some form of acoustic challenge (Mattys et al. 2012) such as background noise or when the speech signal itself is degraded.

Acoustically challenging listening conditions lead to increased cognitive demand (Brungart 2001; Peelle 2018), especially in populations with hearing impairments (Healy and Yoho 2016; Rönnberg et al. 2022). For example, processing moderately degraded speech (such as noise‐vocoded speech with partial intelligibility) has been associated with enhanced BOLD activity in ‘higher‐order’ auditory and speech‐processing regions beyond the superior temporal cortex. Prior studies report that challenging speech engages areas involved in speech articulation and effortful listening—particularly the left precentral gyrus, dorsal anterior insula and inferior frontal gyrus (IFG)—relative to more easily understandable clear speech conditions (Alain et al. 2018; D'Ausilio, Craighero, and Fadiga 2012; D'Ausilio, Bufalari, et al. 2012; Hervais‐Adelman et al. 2012; Wild et al. 2012). Such frontal and insular activations have been interpreted as reflecting articulatory‐based rehearsal or top‐down mechanisms to assist comprehensions under difficult listening conditions (Mattys et al. 2012). Even with hearing protection, the continuous 100 dB background sound during conventional EPI scans can degrade task performance and attention (Brungart 2001; Ke et al. 2021; Schlittmeier and Marsh 2021). Critically, it also introduces confounding brain activity unrelated to the stimuli of interest. The loud constant sound of the scanner can mask stimulus‐evoked auditory responses and induce its own patterns of brain activation, thereby obscuring or altering experimental results (Bandettini et al. 1998; Elliott et al. 1999; Hall et al. 1998; Yakunina et al. 2015). For instance, exposure to typical EPI noise during ‘silent’ rest has been shown to decrease functional connectivity in auditory and sensorimotor networks compared to truly quiet conditions (Rönnberg et al. 2022). Pellegrino et al. (2022) observed reduced connectivity within and between several resting‐state networks (including auditory and language networks) when EPI noise was present, suggesting that standard fMRI noise uniquely perturbs brain dynamics (D'Ausilio, Craighero, and Fadiga 2012; D'Ausilio, Bufalari, et al. 2012; Hervais‐Adelman et al. 2012). Similarly, Yakunina et al. (2015) reported that continuous scanning noise affected activation in not only auditory cortices but also attention and sensorimotor networks during auditory tasks, relative to a sparse acquisition (Alain et al. 2018; Schlittmeier and Marsh 2021). Thus, scanner noise may broadly influence neural activity, extending beyond classical auditory areas and potentially confounding contrasts that seek to isolate responses to auditory stimuli (Elliott et al. 1999; Ke et al. 2021). Moreover, in the context of degraded speech experiments, the scanner's acoustic noise may further degrade or mask the speech stimuli, unintentionally increasing task difficulty or altering the listener's perceptual experience (Peelle 2018). This underscores the need for fMRI methods that minimise acoustic noise, especially for studies of auditory cognition and speech processes.

A common strategy to mitigate scanner noise in auditory fMRI is sparse temporal sampling, where image acquisition is interleaved with silent periods (Hall et al. 1998, 1999; Talavage and Hall 2012). In a sparse‐sampling design, the scanner remains quiet while the auditory stimulus plays, and the volume acquisition is delayed to coincide with the peak of the evoked hemodynamic response (several seconds post stimulus presentation). This approach preserves stimulus audibility and largely avoids simultaneous scanner noise, improving detection of auditory‐evoked activity. However, sparse designs also entail trade‐offs: they substantially reduce sampling rate and temporal resolution and often require longer overall scan times to collect the same amount of data (Talavage and Hall 2012; Yang et al. 2016). Long repetition times (TRs) in sparse fMRI mean fewer volumes per unit time and lower statistical power compared to continuous acquisitions (Nebel et al. 2005; Perrachione and Ghosh 2013; Yakunina et al. 2016). Thus, while effective in enhancing responses to auditory stimulation, sparse sampling sacrifices some sensitivity and flexibility in experimental design.

In recent years, alternative near‐silent MRI sequences have been developed to address these challenges. Notably, sequences based on zero echo time (ZTE) MRI minimise gradient switching by employing a constant readout gradient and non‐cartesian (often radial) k‐space trajectories, enabling virtually silent scanning (Ljungberg et al. 2021). One such sequence, Looping Star (LS), was introduced as a fast and quiet fMRI method operating at only ~2.7 dB above in‐bore ambient noise (Wiesinger et al. 2019; Wiesinger and Solana 2024). LS is a 3D radial multi‐gradient‐echo sequence derived from ZTE techniques, featuring a time‐multiplexed gradient refocusing scheme that yields a free‐induction decay (FID) image followed by multiple gradient echoes (Wiesinger and Solana 2024). The name ‘Looping Star’ reflects its self‐refocusing k‐space trajectory (looping gradients) and radial (star‐like) readout pattern. By avoiding the abrupt gradient switching of EPI, LS dramatically reduces acoustic noise while still sampling the BOLD signal efficiently (Ljungberg et al. 2021; Wiesinger and Solana 2024). Previous studies have demonstrated that LS fMRI can achieve BOLD sensitivity comparable to conventional EPI in a variety of tasks. For example, Wiesinger et al. (2019) showed that an early LS implementation performed similarly to EPI during motor activation tasks. More recently, LS has been validated in cognitive paradigms and resting‐state networks, showing equivalent activation patterns to EPI (Dionisio‐Parra et al. 2020). Critically for auditory research, an initial evaluation of ‘silent’ LS fMRI by Damestani et al. (2021) found that LS detected the expected auditory and higher‐order brain responses while participants discriminated novel sounds in an oddball task without scanner noise interference. These prior results suggest that near‐silent LS can successfully capture task‐related BOLD signals while eliminating confounds caused by acoustic scanner noise.

The present study extends this work by investigating whether LS fMRI maintains sensitivity to brain activations during passive listening to degraded speech, and whether it reveals enhanced responses under adverse listening conditions that might be missed by standard EPI. Unlike previous LS studies that focused on non‐auditory tasks or simple auditory oddball designs, here we directly compare LS and EPI in a speech comprehension context. We hypothesised that multi‐echo LS, by virtue of its near‐silent acquisition and combined echoes, would show at least equivalent activation to EPI for clearly intelligible speech, and greater activation for moderately degraded speech (which requires more effort to understand). We also explored whether adding scanner‐like noise to the stimuli would differentially affect brain responses in the quiet LS environment. To our knowledge, this is the first fMRI study using a near‐silent sequence to examine the neural processing of acoustically degraded speech. By minimising scanner noise, our goal was to reveal the ‘true’ neural correlates of challenging speech perception, and to evaluate LS as a promising tool for auditory cognitive neuroscience.

## 2 Methods

### 2.1 Participants

Ten adult native German speakers (7 female; mean age = 31.2 ± 3.99, age range = 26–38) were recruited from acquaintances and through the study blackboard of the UZH neurolinguistics lab website. Inclusion and exclusion criteria included: 18–40 years of age, no current or past neurological or psychiatric diagnoses, healthy hearing (explicitly stated as a necessity for inclusion on the recruitment flyer and confirmed by participant report), and fluency in the German language, including multilingual persons. Additionally, standard MRI inclusion criteria had to be fulfilled: no metal implants, no large tattoos in the chest, head, or shoulder areas and no dental implants. This study was approved by the local ethics committee (Kantonale Ethikkommission Zürich, BASEC‐Nr. 2022‐0119).

### 2.2 Materials and Experimental Design

**FIGURE 1:**
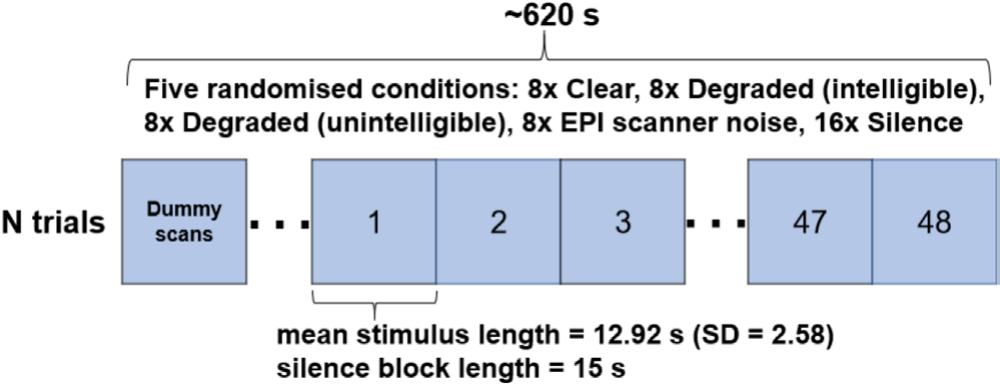
Graphical representation of the experimental paradigm used during the EPI and LS sequences. Participants were scanned twice using EPI and twice using LS. Sequence order was alternated between subjects such that when one subject was scanned using EPI first, the subsequent subject underwent the LS scan first. Each block paradigm consisted of 48 randomised trials: 8 trials of clear speech, 8 trials of intelligible degraded speech, 8 trials of unintelligible degraded speech, 8 trials of clear speech mixed with EPI scanner noise, and 16 trials of silence.

Stimuli were derived from a recording of *Alice in Wonderland* in German (spoken by a female native speaker) (Malik‐Moraleda et al. 2022). We prepared three speech conditions of varying intelligibility plus a control EPI‐like scanner noise condition. From the original audio, 96 short story snippets (mean duration 12.92 ± 2.58) were extracted at sentence boundaries. These were divided into blocks such that each participant heard 32 unique snippets per scan run (eight per condition, with no repeats of intelligible snippets across runs). The conditions were: (1) Clear speech: unaltered snippets (fully intelligible); (2) Moderately degraded speech: snippets processed with a 16‐channel noise vocoder to retain partial intelligibility (~50% keyword transcription accuracy in unpublished pilot testing); (3) Unintelligible speech: snippets noise‐vocoded to be completely unintelligible (extreme spectral degradation); (4) EPI‐like scanner noise: clear speech snippets mixed with recorded EPI scanner noise (signal‐to‐noise ratio reduced by 20 dB to mimic sound levels under ear defenders, noise was recorded during an EPI scan using a Scarlett CM25 MkIII microphone and a Scarlett 2i2 3rd generation audio interface). In addition, silent trials (no auditory stimulation) were included as baseline. Each scan run consisted of 48 trials: eight trials of each of the four auditory conditions and 16 silent trials, presented in pseudorandom order (in a manner such that overlapping stimuli between blocks were always presented in unintelligible form first; lists created with a python script written for this purpose). Stimulus order was read in from pre‐randomised lists and subsequently presented at 65 to 70 dB adjusted peak. Psychopy (version 2022.2.5 (Peirce et al. 2019)) was used to control stimulus presentation and to log stimulus onsets and durations. Trials were separated by brief silent periods to allow hemodynamic responses to rise and fall. Figure 1 shows a graphical representation of the experimental paradigm.

### 2.3 Stimulus Preparation

For the creation of the degraded auditory stimuli, we utilised noise‐vocoding. Noise‐vocoding is a technique that manipulates the spectral properties of speech while maintaining temporal envelope cues (Shannon et al. 1995), allowing for the precise modulation of the severity of information degradation and therefore intelligibility of the content of speech. Using a 16‐channel pulse‐spreading harmonic‐complex vocoder (Mesnildrey et al. 2016), stimuli were spectrally degraded to create moderately intelligible stimuli (yielding keyword transcription performance of approximately 50% in unpublished online pilot tests on *N* = 140 participants) and completely unintelligible stimuli in addition to the clearly understandable stimuli. Additionally, noise from the same EPI sequence used in this study was used to create stimuli mimicking the signal‐to‐noise ratio of a stimulus presented in the EPI scan in the LS runs by mixing the clear stimuli with the recording at a reduced level of −20 dB to account for the attenuating effect of the ear defenders (Model HP‐VS 03/SC 03, CAMBRIDGE RESEARCH SYSTEMS) applied during the scans.

### 2.4 MRI Data Acquisition

MRI data were acquired at the University Children's Hospital, Zürich, Switzerland using a 3 T SIGNA Premier whole‐body MRI scanner equipped with a 48‐channel receive‐only head coil and a body transmit coil. A short 120 s inversion‐recovery T1‐weighted scan (3D T1 BRAVO) with 1 mm isotropic full brain coverage was acquired for anatomical referencing.

The functional imaging protocols were maximally matched between sequences in terms of spatial and temporal resolution parameters, but due to the marked differences in k‐space trajectories between EPI and LS, we expect a wider point‐spread function for LS, leading to a difference in effective resolution (by a factor of 1.25–1.3). This difference is minimized by the spatial smoothing employed during preprocessing, but slight differences may nevertheless persist. The experimental protocol included two runs of a standard EPI sequence (single echo, TE = 28.7 ms, TR = 3.48 s, FOV = 240 mm, slice thickness = 2 mm, number of slices = 54, slice‐gap = 0.5 mm, resolution = 2.5 × 2.5 × 2.5 mm, flip‐angle = 83°, number of volumes = 182) and two runs of the novel LS sequence (coherence resolved version (Ljungberg et al. 2021), multi‐echo, TE0 = 0 ms, TE1 = 14.336 ms, TE2 = 28.672 ms, TR = 3.48 s, FOV = 240 mm, resolution = 2.5 × 2.5 × 2.5 mm, flip‐angle = 2°, pixel bandwidth = 976.562 Hz/pixel, number of volumes = 182, spokes per loop = 16.3, matrix = 100 × 100 × 100, spokes per volume = 1800, bandwidth = 41 KHz, reconstruction = nearest neighbour gridding (Wiesinger et al. 2019)). We did not acquire multi‐echo EPI images due to technical limitations: such a sequence would not have allowed for the precise adjustment of echo times on our scanner setup and therefore would have made it impossible to match the parameters of the LS sequence. Each functional run lasted approximately 620 s. The first three volumes of each run were discarded to ensure steady state magnetisation.

### 2.5 fMRI Preprocessing

**FIGURE 2:**
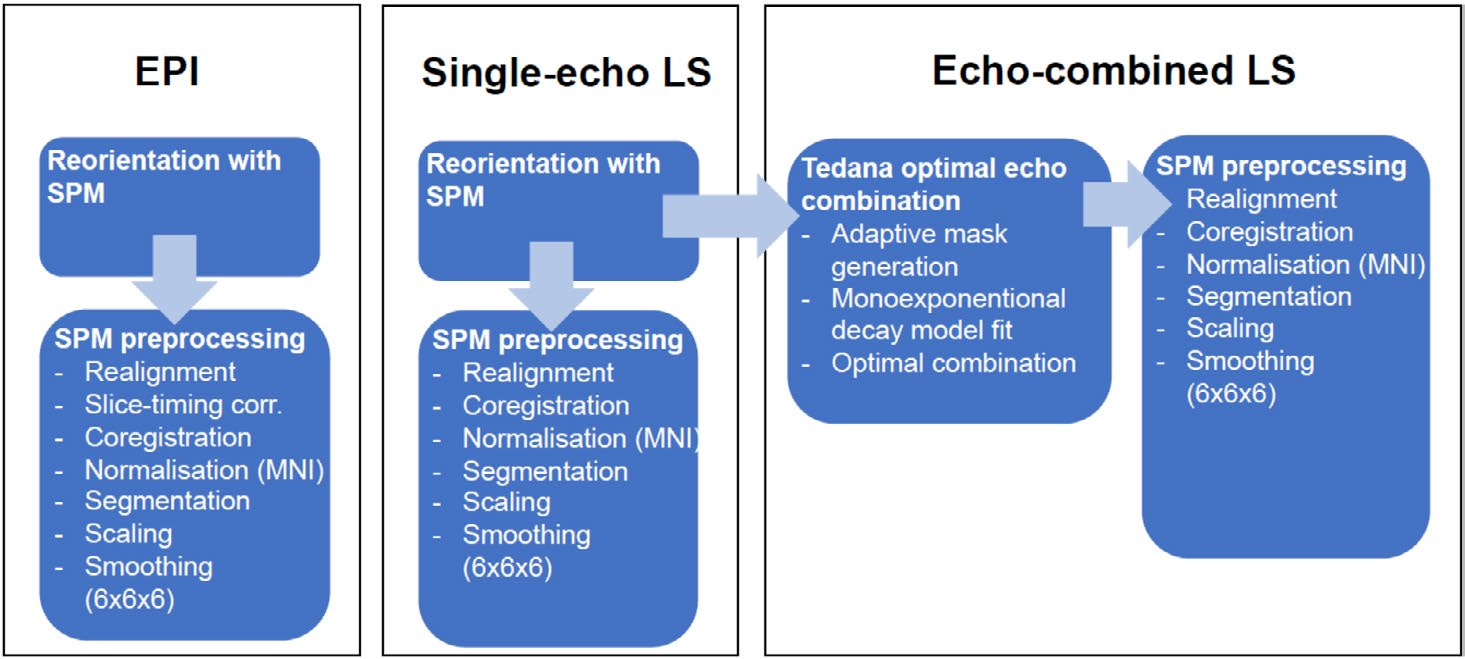
Graphical representation of the pre‐processing pipeline.

Functional and anatomical images were preprocessed using SPM12 (7771) (Wellcome Centre for Human Neuroimaging, London; https://fil.ion.ucl.ac.uk/spm/) with identical steps for both EPI and LS data, except where noted. The multi‐echo LS data were first combined across echoes to leverage the sequence's full signal. We used the *tedana* Python library (ME‐ICA v23.0.1 (Ahmed et al. 2023)) to perform optimal echo combination on the LS acquisitions. During this procedure, a weighted average of the echoes based on their temporal SNR is created, which maximises overall BOLD sensitivity (DuPre et al. 2021; Kundu et al. 2013, 2012). As the TE = 0 ms echo shows no BOLD contrasts, its weight during the echo combination is effectively zero. This yielded two versions of LS data: a combined multi‐echo time series and a single‐echo time series (using only the second echo, TE2 = 28.67 ms) for comparison with EPI. Each participant's dataset (EPI, LS single‐echo, LS echo‐combined) then underwent standard preprocessing. First, images were manually reoriented with the origin set at the anterior commissure. Slice timing correction: Performed only for EPI, as LS used a 3D readout. Realignment: Functional volumes were motion‐corrected by realigning to the first image of each run. Coregistration: The mean functional image from each sequence was coregistered to the high‐resolution T1 anatomical image. Segmentation and Normalisation: The T1 image was segmented into tissue classes and used to compute transformation parameters to MNI standard space, these parameters were applied to warp all functional images to MNI space at 2 mm isotropic resolution. Smoothing: All functional images were spatially smoothed with a 6 mm full‐width at half‐maximum Gaussian kernel. Figure 2 provides a schematic of the preprocessing pipeline.

### 2.6 Statistical Analysis

#### 2.6.1 SPM Within‐ and Between‐Sequence Analyses

Using the onsets and durations of stimulus blocks logged in Psychopy (Peirce et al. 2019), we fit a GLM specifying the conditions. The silent blocks were left unmodelled and served as an implicit baseline for subsequent analyses. Motion parameters extracted from the realignment step in SPM were added as covariates in the first‐level model specification. After model estimation, contrast files were created comparing each of the experimental conditions to the baseline as well as each other.

These first‐level contrast files were then used in second‐level non‐parametric analyses utilising SnPM13.1.09 (https://www.nisox.org/Software/SnPM13/). We conducted one‐sample *t*‐tests to assess within‐sequence BOLD differences between the stimulus conditions and paired *t*‐tests to test for between‐sequence effects using non‐parametric permutation tests. To construct the empirical null distribution, 1024 permutations were used (possible number of permutations = 2^n^, where n is the number of subject data). Results were thresholded at an initial voxel‐level *t*‐threshold of 3.09 (corresponding to *p* = 0.001, uncorrected) and then at the cluster level with a family‐wise error (FWE) corrected threshold of *p* < 0.05. We also conducted variance smoothing to reduce uncertainty of the variance estimate. Due to variance smoothing, pseudo‐*t* values are reported rather than *t*‐values. Non‐parametric analyses were chosen due to the low group size of *n* = 10 and resulting low degrees of freedom.

#### 2.6.2 Between‐Sequence Temporal Signal‐To‐Noise Ratio Analysis

Additionally, we compared temporal signal‐to‐noise ratio (tSNR) values (averaged over participants) between sequences in the same manner. tSNR values were extracted using an in‐house Matlab script calculating the mean voxel values divided by their standard deviation in the time series of the raw functional images for each voxel in the image (in accordance with Dionisio‐Parra et al. (2020)). This calculation was carried out for each of the subjects and sessions acquired for EPI, single‐echo LS and echo‐combined LS. Sequence‐specific tSNR maps were subsequently averaged and compared using Kruskal‐Wallis tests in R version 4.3.1.

#### 2.6.3 Reliability Assessment

To assess reliability within and between sequences, we conducted voxel‐wise intra‐class correlation (ICC) analysis within and between EPI and echo‐combined LS (Caceres et al. 2009). ICC index (3,1) (Shrout and Fleiss 1979) was used to assess between‐session reliability for the contrast maps of Sound > baseline (ICC between scan 1 and scan 2 for each modality), as it has been proposed for exactly this kind of comparison. The ICC (3,1) calculates the ratio of the sum of squares between participants (SBP) and that between sessions (SBS), with k as the number of repeated sessions:

$$\mathrm{ICC}\left(3,1\right)=\frac{\mathrm{SBP}-\mathrm{SBS}}{\mathrm{SBP}+\left(k-1\right)\cdot \mathrm{SBS}}$$

This yields ICC values between −1 and 1, with values closer to 1 indicating high between‐session reliability and a near‐zero sum of squares ratio between sessions. See also (Damestani et al. 2021) for a similar approach.

Contrast maps for the Sound > baseline condition for each participant and each session (scan 1 and scan 2) were used to conduct these analyses. All analyses were conducted using the SPM reliability toolbox (Pernet 2016).

In a first step, we calculated ICC *z*‐scores between Scan 1 and Scan 2 across the whole‐brain for each of the sequences separately. The resulting maps and all other results of the reliability analyses can be seen in Figure 6. The *z*‐scores of each of the voxels from this first analysis were extracted and used to create relative voxel frequency plots as well as joint distribution plots of the ICC scores versus the corresponding *t*‐scores.

In the next step, the contrast files were masked with the SPM grey matter prior. We then extracted the voxel values from each participant and for each scan within an auditory ROI extracted from Neurosynth (using the search term ‘auditory’, thresholded at *z* = 5) (Yarkoni et al. 2011) and plotted these against each other. Subsequently, intra‐voxel reliability (ICCv) within this auditory ROI was calculated between sessions for each participant and each sequence. This yields an ICC value for the whole region masked with the ROI mask, rather than a value for each voxel within the mask. This resulted in one intra‐voxel ICCv value for each participant, which was then compared between sequences using a two‐tailed *t*‐test with (sig. *p* < 0.05). For this, the above stated equation is applied for each participant using the contrast values of the voxels within the applied mask (Caceres et al. 2009). The participant‐specific ICCv measures the variance which is explained by intra‐voxel variance. Between‐subject differences are inferred by testing the consistency of the spatial distribution of the BOLD signal.

## 3 Results

We report only significant results in the following section; for a complete overview of assessed contrasts see Table 1.

**TABLE 1:** Overview of contrast which were assessed during statistical analysis.

	Comparison	Assessed in or between the following sequences	Result of comparison
Within‐sequence One‐sample *t*‐tests	All sound > baseline	EPI	Significant
		Single‐echo LS	Significant
		Echo‐combined LS	Significant
	Clear > baseline	EPI	Significant
		Single‐echo LS	Significant
		Echo‐combined LS	Significant
	Moderately intelligible > baseline	EPI	Significant
		Single‐echo LS	Significant
		Echo‐combined LS	Significant
	Clear > moderately intelligible	EPI	Significant
		Single‐echo LS	Significant
		Echo‐combined LS	Significant
	Moderately intelligible > clear	EPI	Non‐significant
		Single‐echo LS	Non‐significant
		Echo‐combined LS	Non‐significant
	Scanner noise > clear	Single‐echo LS	Non‐significant
		Echo‐combined LS	Non‐significant
	Clear > scanner noise	Single‐echo LS	Non‐significant
		Echo‐combined LS	Non‐significant
Between‐sequence Paired *t*‐tests	All sound > baseline	Single‐echo LS versus EPI	Non‐significant
		Echo‐combined LS versus EPI	Significant
		Echo‐combined LS versus Single‐echo LS	Non‐significant
	Clear > baseline	Single‐echo LS versus EPI	Non‐significant
		Echo‐combined LS versus EPI	Significant
		Echo‐combined LS versus Single‐echo LS	Non‐significant
	Moderately intelligible > baseline	Single‐echo LS versus EPI	Non‐significant
		Echo‐combined LS versus EPI	Significant
		Echo‐combined LS versus Single‐echo LS	Non‐significant
	Clear > moderately intelligible	Single‐echo LS versus EPI	Non‐significant
		Echo‐combined LS versus EPI	Significant
		Echo‐combined LS versus Single‐echo LS	Non‐significant
	Moderately intelligible > clear	Single‐echo LS versus EPI	Non‐significant
		Echo‐combined LS versus EPI	Non‐significant
		Echo‐combined LS versus Single‐echo LS	Non‐significant
	Scanner noise > clear	Single‐echo LS versus EPI	Non‐significant
		Echo‐combined LS versus EPI	Non‐significant
		Echo‐combined LS versus Single‐echo LS	
	Clear > scanner noise	Single‐echo LS versus EPI	Non‐significant
		Echo‐combined LS versus EPI	Non‐significant
		Echo‐combined LS versus Single‐echo LS	

*Note:* Comparisons depicted as significant reached a cluster‐level FWE corrected *p* value < 0.05 or lower.

### 3.1 Within‐Sequence One‐Sample t‐Tests

Within‐sequence second‐level cluster‐wise one‐sample *t*‐tests between the contrasts modelled in the first‐level analysis showed similar BOLD activation for each sequence (EPI, single echo‐LS and echo‐combined LS) mostly centered around the bilateral middle and superior temporal gyri. In these regions, EPI and single‐echo LS exhibited similar cluster sizes, whereas echo‐combined LS consistently showed larger clusters (in terms of number of voxels).

**FIGURE 3:**
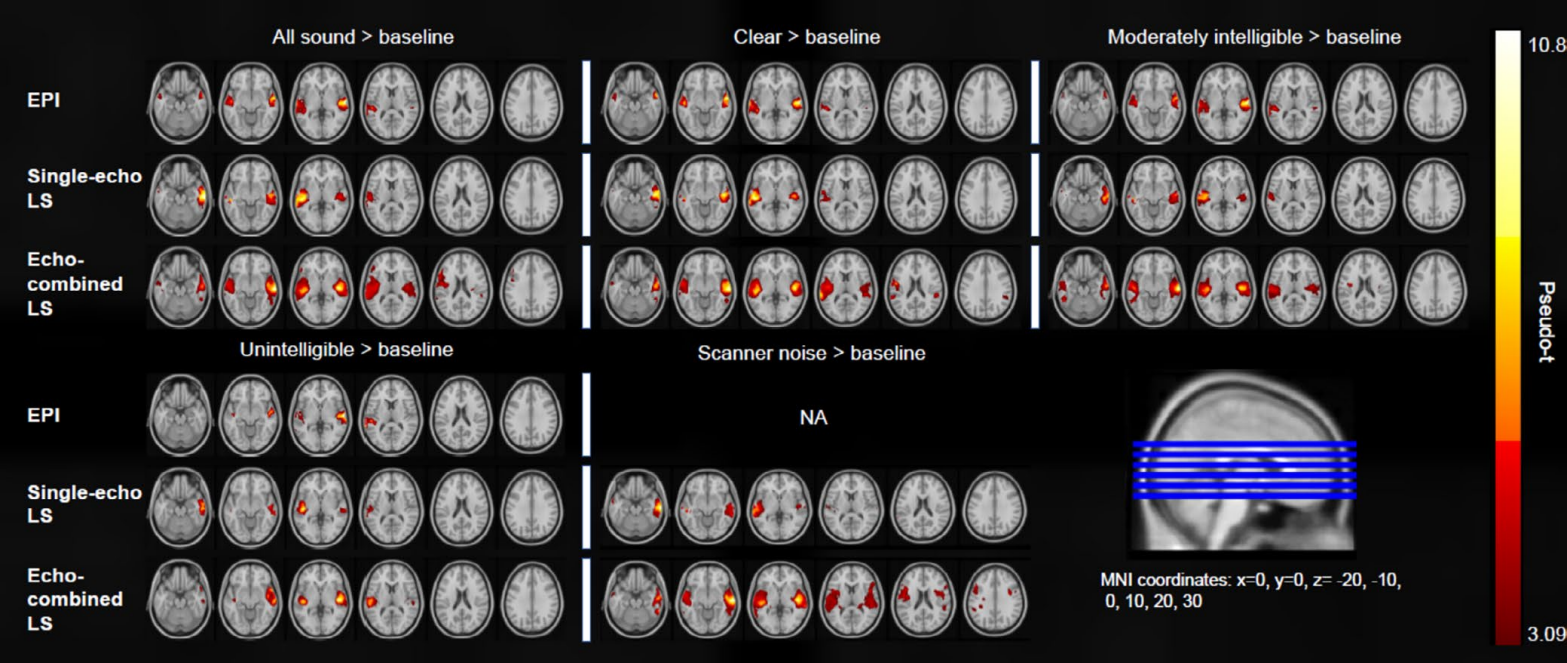
Results of second‐level within‐sequence one‐sample *t*‐tests for the conditions versus baseline in EPI, single‐echo LS and echo‐combined LS. One‐sample t‐tests were calculated using SnPM (*p* < 0.05 (FWE‐corrected at cluster level), clusterforming threshold = 3.0902). Only significant comparisons in at least one sequence are depicted.

**FIGURE 4:**
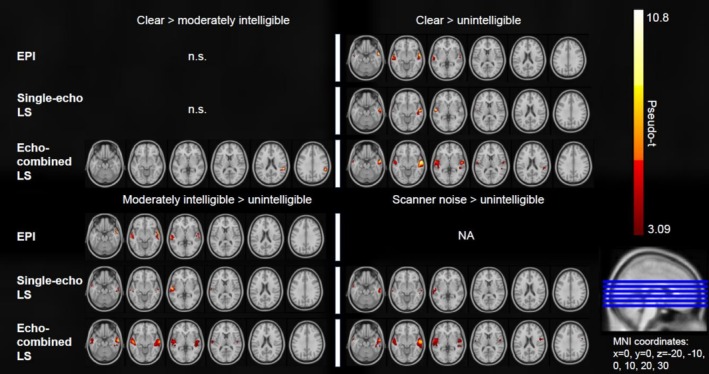
Results of second‐level within‐sequence one‐sample t‐tests for comparisons between conditions in EPI, echo‐combined LS and single‐echo LS. One‐sample t‐tests were calculated using SnPM (*p* < 0.05 (FWE‐corrected at cluster level), cluster forming threshold = 3.0902). Only significant comparisons in at least one sequence are depicted.

Only echo‐combined LS showed significant peak voxels in brain areas outside of the bilateral middle and superior temporal gyri. In the following paragraphs, we describe these significant BOLD clusters located outside of the middle and superior temporal gyri found in the within‐sequence analysis for echo‐combined LS. For an overview of the significant within‐sequence comparisons see Figures 3 and 4 as well as Tables 2 and 3.

**TABLE 2:** Within‐sequence one‐sample *t*‐tests. Comparison of experimental conditions with the implicit baseline condition.

Comparison	Sequence	AAL3 brain region	Peak voxel coordinates (MNI x, y, z [in mm])	Peak pseudo‐t	Cluster size (*n* voxels)
All sound > baseline	EPI	R. STG., post. div.	62, −14, 0	7.99**	1695
		R. STG., ant. div.	56, ‒6, −8	6.68**	2155
		R. STG., post. div.	52, −14, −4	6.63**	
		L. STG, post. div.	−64, −18, 4	5.75**	
	LS single echo	L. STG, plan. pol.	−44, −18, −4	7.31**	2187
		L. MTG, post. div.	−54, −26, −4	7.04**	2645
		L. MTG, post. div.	−52, −18, −6	6.64**	
		R. MTG, post. div.	62, −18, −20	7.18**	
		R. MTG, post. div.	60, −8, −24	6.02**	
	Echo‐combined LS	R. MTG, post. div.	64, −18, −12	9.71**	5237
		R. STG, post. div.	48, −18, −2	8.59**	6608
		L. STG, plan. temp.	−48, −30, 4	7.12**	
		L. IFG., pars op.	−56, 20, 20	6.33**	
		L. MTG, post. div.	−64, −34, 6	5.9**	
Clear > baseline	EPI	R. STG, post. div.	64, −14, −2	7.55**	1648
		R. STG, ant. div.	56, −6, −8	6.79**	1933
		R. MTG, ant. div.	58, 2, −20	6.01**	
		L. STG, plan. temp.	−64, −18, 4	5.85**	
		L. MTG, ant. div.	−62, −10, −6	5.3**	
	LS single echo	L. MTG, post. div.	−54, −24, −4	7.05**	1917
		L. MTG, post. div.	−66, −34, 2	5.36**	2363
		R. ITG, post. div.	50, −16, −22	6.15**	
		R. STG, post. div.	50, −16, −8	6.01**	
		R. MTG, post. div.	58, −14, −18	5.94**	
	Echo‐combined LS	R. MTG, post. div.	62, −18, −12	8.48**	5023
		R. STG, post. div.	50, −16, −2	7.12**	5162
		R. MTG, ant. div.	62, −4, −16	6.08**	
		L. STG, post. div.	−64, −36, 6	7.32**	
		L. MTG, post. div.	−48, −28, 2	6.82**	
		L. PostCG.	−50, −6, 18	5.59**	
Moderately intelligible > baseline	EPI	R. STG, post. div.	62, −14, 0	8.63**	1918
		R. STG, post. div.	50, −14, −4	7.07**	1985
		R. STP	56, 8, −14	5.51**	
		L. STG, plan. temp.	−64, −18, 4	5.42**	
	LS single echo	L. STG, plan. pol.	−42, −16, −6	9.86**	1912
		L. MTG, post. div.	−50, −14, −6	7.43*	2272
		L. MTG, post. div.	−58, −24, −4	7.09**	
		R. MTG, post. div.	62, −14, −16	6.65**	
		R. ITG, post. div.	54, −24, −20	6.45**	
		R. MTG, post. div.	64, −16, −24	6.36**	
	Echo‐combined LS	R. MTG, post. div.	64, −18, −12	10.76**	4369
		R. STG, post. div.	48, −18, −4	8.17**	4858
		L. STG, plan. pol.	−40, −20, −2	6.79**	
		L. STG, prim. aud.	−44, −28, 4	6.61**	
Unintelligible > baseline	EPI	R. STG, post. div.	64, −12, −2	7.18**	1080
		R. STG, post. div.	50, −14, −6	6.08**	958
		R. STG, post. div.	66, −24, 2	5.57**	
		L. STG, post. div.	−64, −42, 8	5.43**	
	LS single echo	L. STG, plan. pol.	−44, −18, −4	6.78**	1154
		R. MTG, post. div.	60, −18, −18	5.41*	1323
	Echo‐combined LS	R. STG, post. div.	50, −16, −2	6.65**	2613
		L. STG, Heschl's G. (H1 and H2)	−48, −24, 4	5.38*	1554
Scanner noise > baseline	EPI	—	—	—	—
	LS single echo	R. MTG, post. div.	64, −18, −18	8.19**	2072
		L. MTG, post. div.	−54, −24, −6	6.79**	1515
		L. MTG, post. div.	−48, −30, −6	5.81**	
		L. MTG, ant. div.	−62, −10, −12	5.7**	
	Echo‐combined LS	R. STG/MTG, post. div.	64, −18, −10	10.11**	7032
		*R. STG, plan. pol.	46, −16, −2	9.01**	6915
		R. ITG, temp.occ.	58, −44, −20	6.42**	
		L. STG, plan. temp.	−46, −28, 2	6.92	

*Note:* Pseudo‐*t* contrast after variance smoothing, calculated using SnPM. *p < 0.05, FWE corrected at the cluster level, **p < 0.05, FWE corrected at the voxel level.

Abbreviations: ant./post div., anterior/posterior division; IFG, inferior frontal gyrus; ITG, inferior temporal gyrus; MTG, middle temporal gyrus; pars. op., pars opercularis; plan. pol., planum polare; plan. temp., planum temporale; PostCG, postcentral gyrus; STG, superior temporal gyrus.

**TABLE 3:** Within‐sequence one‐sample *t*‐tests.

Comparison	Sequence	AAL3 brain region	Peak voxel coordinates (MNI x, y, z [in mm])	Peak pseudo‐t	Cluster size (*n* voxels)
Clear > moderately intelligible	EPI	n.s.	n.s.	n.s.	n.s.
	LS single echo	n.s.	n.s.	n.s.	n.s.
	Echo‐combined LS	R. SMG, post. div.	56, −38, 24	4.78*	443
Clear > unintelligible	EPI	R. MTG, ant. div.	58, 2, −20	7.37**	718 679
		R. STG, post. div.	64, −12, −6	5.62**	
		L. MTG, post. div.	−58, −26, −6	5.15*	
	LS single echo	L. STG, ant. div. R. MTG, post. div.	−54, −8, −4 64, −12, −16	5.69** 4.9*	395 772
	Echo‐combined LS	R. MTG, post. div.	56, −10, −14	6.59**	1881 1217
		L. MTG, ant. div.	−60, −8, −6	4.4*	
Moderately intelligible > unintelligible	EPI	R. STG, ant. div.	58, 4, −14	5.84**	745 561
		R. STG, post. div.	64, −14, −6	5.39**	
		L. STP	−56, 4, −12	4.93*	
	LS single echo	L. STG, ant. div.	−52, −10, −6	7.91**	640 391
		R. MTG, post. div.	64, −12, −14	5.07*	
	Echo‐combined LS	R. MTG, post. div.	66, −14, −14	9.41**	2166 1685
		L. MTG, post. div.	−52, −18, −10	6.22**	
Scanner noise > unintelligible	EPI	—	—	—	—
	LS single echo	R. MTG, post. div.	66, −16, −16	6.32**	429
		L. MTG, ant. div.	−62, −6, −12	5.04*	485
	Echo‐combined LS	R. MTG, post. div.	64, −8, −16	9.26**	2327
		L. STG, ant. div.	−60, −6, −2	5.76**	1449

*Note:* Comparison between experimental conditions. Pseudo‐*t* contrast after variance smoothing, calculated using SnPM. *p < 0.05, FWE corrected at the cluster level, **p < 0.05, FWE corrected at the voxel level.

Abbreviations: ant./post div., anterior/posterior division; MTG, middle temporal gyrus; SMG, supramarginal gyrus; STG, superior temporal gyrus; STP, superior temporal pole.

#### 3.1.1 All Sound > Baseline

In the comparison between all Sound > baseline, we found a significant sub peak activation in the left inferior frontal gyrus (IFG), pars opercularis within a large cluster spanning the left precentral gyrus, the inferior frontal gyrus, pars opercularis and the inferior frontal gyrus, pars triangularis (peak pseudo‐*t* = 6.33, peak *p*_(FWE‐corr)_ < 0.001, cluster size = 6608, MNI_(x,y,z in mm)_ = −56, 20, 20).

#### 3.1.2 Clear > Baseline

In the Clear > baseline condition, a significant sub peak activation was revealed located at the left central sulcus between the post‐ and precentral gyri (peak pseudo‐*t* = 5.59, peak *p*_(FWE‐corr)_ < 0.001, cluster size = 5162, MNI_(x,y,z in mm)_ = −50, −6, 18). This cluster also extended into the left frontal lobe, specifically the inferior frontal gyrus, pars opercularis and the precentral gyrus.

#### 3.1.3 Clear > Moderately Intelligible

For the within‐sequence between‐condition comparisons, the Clear > moderately intelligible comparison showed a significant peak voxel activation in the right supramarginal gyrus, posterior division (peak pseudo‐t = 4.78, cluster *p*_(FWE‐corr)_ < 0.001, cluster size = 443, MNI_(x,y,z in mm)_ = 56, −38, 24). The cluster in which this peak voxel was embedded also extended into the right superior temporal cortex. Note that none of the other sequences except for echo‐combined LS showed any significant differences in activation in this comparison.

### 3.2 Between‐Sequence Paired *t*‐Tests

**FIGURE 5:**
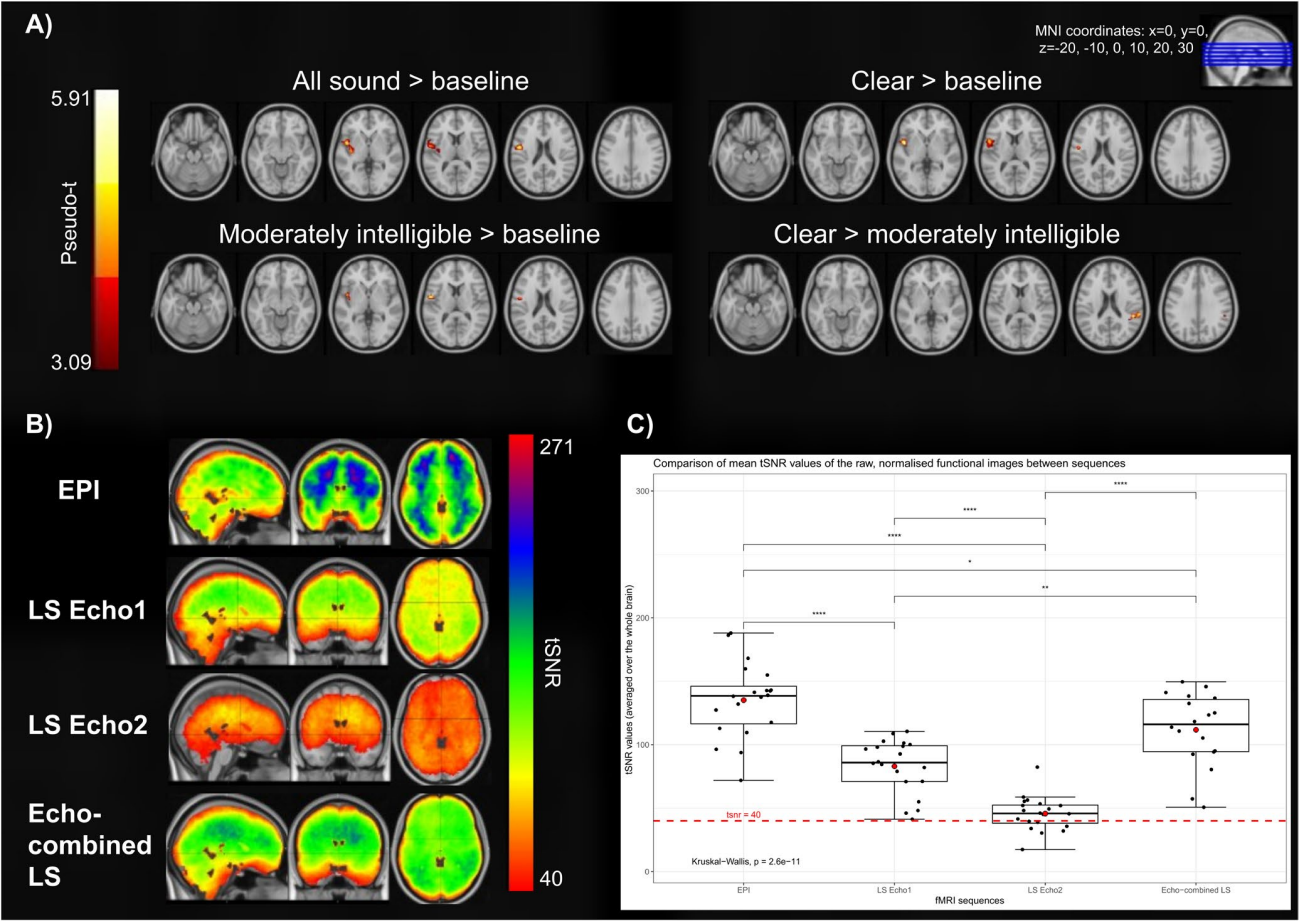
(A) Results of second‐level between‐sequence paired *t*‐tests of cluster‐level BOLD differences for the comparison echo‐combined LS versus EPI. Other comparisons were non‐significant. Paired *t*‐tests were calculated using SnPM (*p* < 0.05 (FWE‐corrected at cluster level), clusterforming threshold = 3.0902). (B) A depiction of tSNR values for each sequence, averaged over the functional images of each participant and each scan. The lower tSNR threshold of 40 was chosen as this has been identified in previous research as the minimum tSNR at which between condition differences could be reliably detected in fMRI^4^. (C) Boxplot showing results of a Kruskal‐Wallis test and post hoc pairwise Wilcoxon test of average whole‐brain tSNR values between sequences. The box depicts the interquartile range, while the line denotes the median. Red squares denote the mean. Error bars depict the standard error of the mean. * < 0.05, ** < 0.01, *** < 0.001, **** < 0.0001.

Second‐level paired *t*‐tests between the sequences of contrasts modelled in the first‐level analysis showed significant differences for the comparison of echo‐combined LS > EPI. There were no other significant differences when comparing single‐echo LS to EPI or to echo‐combined LS. In the following paragraphs, these significant between‐sequence comparisons (echo‐combined LS > EPI) are reported. For an overview of the significant between‐sequence results see Figure 5a and Table 4.

**TABLE 4:** Paired between‐sequence *t*‐tests.

Comparison	AAL3 brain region	Peak voxel coordinates (MNI x, y, z [in mm])	Peak pseudo‐t	Cluster size (*n* voxels)
All sound > baseline	L. PostCG, primary motor	−54, −6, 16	5.65**	949
	L. ant. insula	−46, 2, 0	4.95*	
	L. post. insula	−36, −14, −2	4.67*	
Clear > baseline	L. ant. insula	−46, 2, 2	4.67*	427
	L. PostCG	−50, −6, 18	4.21	
Moderately intelligible > baseline	L. PreCG, premotor/supp. motor	−54, −4, 12	4.88*	290
	*L. ant. insula	−44, 2, 0	4.31*	
	L. PreCG, premotor/supp. motor	−52, −4, 24	4.05*	
Clear > moderately intelligible	R. STG, post. div.	50, −40, 22	4.62	233
	R. STG, plan. temp.	62, −32, 16	4.31*	
	R. STG, post. div.	60, −38, 22	4.13*	

*Note:* Peak voxels for significant results of the comparison combined‐echo LS > EPI (no other comparisons were significant). Pseudo‐*t* contrast after variance smoothing, calculated using SnPM. *p < 0.05, FWE corrected at the cluster level, **p < 0.05, FWE corrected at the voxel level.

Abbreviations: ant./post., anterior/posterior; plan. temp., planum temporale; post. div., posterior division; PostCG, postcentral gyrus; PreCG, precentral gyrus; STG, superior temporal gyrus.

#### 3.2.1 All Sound > Baseline

Echo‐combined LS showed significantly stronger BOLD activation than EPI in the following clusters when comparing first‐level derived contrast files for the all Sound > baseline. We found a significant left hemisphere cluster extending into the precentral gyrus, the insula, the Rolandic operculum, the postcentral gyrus and the inferior frontal gyrus, with peak activations located in the postcentral gyrus and the anterior and posterior insulae (peak pseudo‐*t* = 5.65, cluster *p*_(FWE‐corr)_ = 0.002, cluster size = 949, MNI_(x,y,z in mm)_ = −54, −6, 16).

#### 3.2.2 Clear > Baseline

In the between‐sequence comparison of Clear > baseline contrast files, a left hemisphere cluster extending into the superior temporal pole, the rolandic operculum, the inferior frontal operculum, the insula, the postcentral gyrus and the precentral gyrus was revealed (peak pseudo‐*t* = 4.67, cluster p_(FWE‐corr)_ = 0.011, cluster size = 427, MNI_(x,y,z in mm)_ = −46, 2, 2). Peak voxel activations were located in the anterior insula and the postcentral gyrus.

#### 3.2.3 Moderately Intelligible > Baseline

The between‐sequence comparison of the Moderately intelligible > baseline contrast files showed a left hemisphere cluster extending into the rolandic operculum, the insula, the postcentral gyrus and the precentral gyrus with peak voxels located in the precentral gyrus and the anterior insula (pseudo‐*t* = 4.88, cluster *p*_(FWE‐corr)_ = 0.039, cluster size = 290, MNI_(x,y,z in mm)_ = −54, −4, 12).

#### 3.2.4 Clear > Moderately Intelligible

The comparison of Clear > Moderately intelligible contrast files between sequences showed a significant right hemisphere cluster extending from the posterior superior temporal gyrus into the supramarginal gyrus with peak activations in the posterior superior temporal gyrus and the planum temporale (pseudo‐*t* = 4.62, cluster *p*_(FWE‐corr)_ = 0.029, cluster size = 233, MNI_(x,y,z in mm)_ = 50, −40, 22) for multi‐echo LS.

### 3.3 Between‐Sequence Comparison of tSNR

We further compared tSNR values between the sequences as an additional indicator of sensitivity. All sequences showed average tSNR's above 40 across the brain, a threshold which has been identified in previous research as the lower level at which between‐condition differences can be reliably detected in fMRI paradigms (Murphy et al. 2007). A Kruskal‐Wallis test with post hoc pairwise Wilcox comparisons showed significantly higher average tSNR in the EPI sequence when compared to single‐echo LS (Echo1: *r* = 0.723, *p* < 0.001, Echo2: *r* = 0.851, p < 0.001), as well as significantly higher tSNR compared to echo‐combined LS (*r* = 0.365, *p* = 0.024) which in turn showed significantly higher tSNR than its single echoes (Echo1: *r* = 0.507, *p* = 0.002, Echo2: *r* = 0.806, *p* < 0.001). For a graphical representation of the tSNR maps for all sequences see Figure 5b.

### 3.4 Reliability Analysis

**FIGURE 6:**
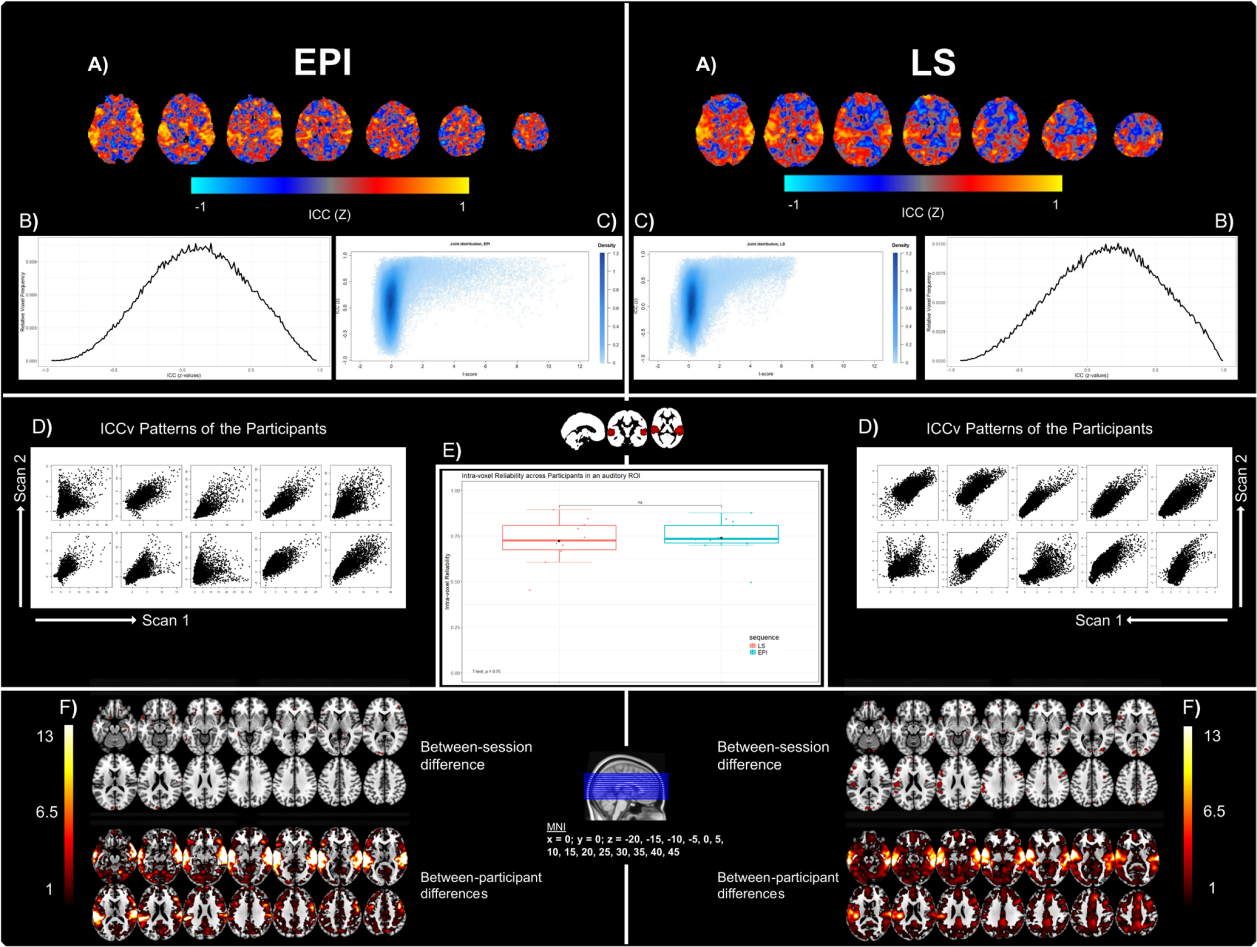
Plots depicting test–retest reliability for the Sound > baseline conditions in EPI and echo‐combined LS. (A) Whole‐brain intra‐class correlation (ICC) *z*‐score maps for each modality. (B) Line diagrams showing the relative voxel frequencies versus ICC *z*‐scores. (C) Plots of ICC coefficient versus the corresponding *t*‐score for that voxel (whole‐brain). (D) Voxel‐wise ICC (ICCv) patterns of Scan 1 versus Scan 2 for each of the participants. The ROI from which these values have been extracted can be seen above E. The mask was created by searching for the search term “auditory” on Neurosynth (Yarkoni et al. 2011), and subsequently thresholding the downloaded brain map at *z* = 5. (E) A box‐and‐whisker plot of the intra‐voxel reliability (concordance correlation coefficient rho) for each participant and for each modality. (F) Whole‐brain between‐session and between‐participant differences for each of the modalities overlaid over the MNI standard brain using MRIcroGL (Rorden and Brett 2000).

In addition to the within‐ and between‐session *t*‐tests an ICC analysis was conducted. As can be seen in Figure 6A, group‐level within‐sequence ICC's were calculated across the whole brain on the contrast maps of the Sound > baseline condition. The resulting ICC maps show consistently high ICC *z*‐values in auditory‐related regions (e.g., superior and middle temporal lobes) for both sequences. Outside of these regions, LS showed larger clusters of negative ICC values, indicating lower reliability in these sections of the brain. Joint distribution analysis shows a larger percentage of voxels with high ICC values (> 0.75) in EPI (19.4%) than in LS (15.1%), and a distribution which was more skewed to higher t‐values in EPI (Figure 6B).

Using an auditory ROI derived from Neurosynth, intra‐voxel reliability (ICCv) was calculated between scan sessions for each participant. When comparing the average ICCv values across participants between sequences, a two‐tailed *t*‐test yielded no significant results (*p* = 0.75). Low ICCv values seen in plot E in Figure 6 were not produced by the same participant across sequences.

An investigation of between‐session and between‐participant sum of squares showed large differences between participants for both sequences. Between‐session differences were more pronounced in the LS sequence which showed clusters of high between‐session variance in the left operculum, left IFG, occipital cortex, middle frontal gyrus, parts of the left supramarginal gyrus (SMG) and parts of the bilateral precentral gyri. EPI, in contrast showed fewer clusters of high between‐session variance located in the frontal orbital cortices, left temporal pole, right fusiform gyrus and the occipital cortex. Both sequences showed high between‐participant variance in the bilateral temporal cortices and lateral cortex areas in general, with LS showing overall higher and more widespread between‐subject variance than EPI.

## 4 Discussion

In the current study, we investigated the sensitivity of a novel LS fMRI in comparison to standard EPI while participants passively listened to speech stimuli of varying intelligibility: clear, moderately degraded, or incomprehensibly degraded speech. LS was selected for this study due to its near‐silent properties, a feature beneficial for auditory neuroscience and especially relevant for studies of the neural processing of degraded speech. In addition, we directly assessed the effect of scanner noise on speech perception within the LS sequence by including a condition in which clear speech was mixed with recorded EPI acoustic noise.

In within‐sequence comparisons across auditory conditions, we consistently observed BOLD signal activation in the bilateral medial and superior temporal gyri for EPI, single‐echo LS and echo‐combined LS across all audio conditions. EPI and single‐echo LS yielded similar activation patterns with respect to cluster locations, sizes and peak pseudo‐t values. In contrast, echo‐combined LS demonstrated consistently larger clusters and higher peak pseudo‐*t* values which extended into brain regions beyond the temporal gyri. A comparison of all auditory conditions (Sound) to the silent baseline in echo‐combined LS revealed a large activation cluster in the left hemisphere with sub‐peak activation in the IFG, specifically in the pars opercularis. This activation cluster also included the pars triangularis of the IFG, the pre‐ and postcentral gyri, the SMG, the Rolandic operculum, as well as the insula. In the right hemisphere, analyses revealed a significant BOLD signal cluster spanning the putamen, the Rolandic operculum as well as the insula whereas all the other sequences showed no activation in these brain areas. These regions are consistent with networks associated with effortful and complex speech processing. The IFG has been shown to be involved during speech processing when perception is challenging (D'Ausilio, Craighero and Fadiga 2012; D'Ausilio, Bufalari, et al. 2012; Hervais‐Adelman et al. 2012). The anteroventral SMG has been implicated in internal speech and speech production (Oberhuber et al. 2016; Wandelt et al. 2024), verbal working memory (Deschamps et al. 2014) and bottom‐up auditory‐to‐motor transformation, including auditory feedback processing and vocal compensation of feedback errors (Li et al. 2022). The Rolandic operculum, particularly the posterior part, may be involved in the processing of prosodic information (Ischebeck et al. 2007) and impaired auditory processing has been linked to Rolandic epilepsy in children (Smith et al. 2017). The insula has been implicated in speech perception and articulatory control (Oh et al. 2014) and been identified as part of the neural network involved in effortful processing, for example in the case of degraded speech perception (Hervais‐Adelman et al. 2012) and may serve as a relay between cognitive and motor aspects of speech (Adank 2012; Oh et al. 2014).

Although directly comparing moderately degraded and clear speech did not yield statistically significant activation in these brain regions related to effortful processing—potentially due to limited sample size and a resulting lack of statistical power—the findings in the Sound > baseline contrast might indicate a heightened sensitivity in echo‐combined LS for these kinds of activation patterns. This is supported by the findings of a distinct cluster of BOLD activation in the left opercular cortex and parts of the left insula in echo‐combined LS for the Moderately degraded > baseline contrast, and the absence of such a finding in EPI as well as in the Clear > baseline contrast within echo‐combined LS. This may suggest that while a direct effect of degradation level on these effort‐related areas was not observed, echo‐combined LS may exhibit greater sensitivity to neural substrates of effortful speech processing. This sensitivity advantage may become more apparent with increased statistical power or additional data.

For a direct assessment of sensitivity between sequences, we conducted cluster‐wise paired *t*‐tests on first‐level contrast images derived from each participant, comparing speech conditions across sequences. The analyses revealed no significant differences in BOLD activation between single‐echo LS and EPI. Although the average whole‐brain tSNR was lower for single‐echo LS compared to EPI, it remained above 40—a threshold previously established as sufficient for reliable BOLD detection (Murphy et al. 2007)—across most brain areas, including regions relevant to (degraded) speech processing, such as the superior temporal gyri, IFG and precentral gyri. These results suggest that single‐echo LS may provide sufficient sensitivity for investigating degraded speech. Comparisons between echo‐combined LS and EPI revealed significantly stronger BOLD signal in multiple regions, alongside significantly lower mean tSNR values in echo‐combined LS. This included Sound > baseline and Moderately degraded > baseline contrasts, where we found significantly stronger BOLD activation in the left anterior insula, precentral gyrus and the IFG. These findings, despite lower sensitivity in echo‐combined LS, may indicate a masking effect of the background noise in EPI and lack thereof in LS. These results should nevertheless be carefully interpreted, as differences in the detected BOLD signal may be significantly confounded by differences in the sequences other than the noiselessness of LS. Additional reliability analyses (ICC) of the Sound > baseline contrast indicated comparable and high reliability between scan sessions in both sequences, despite EPI showing increased numbers of high ICC voxels in the joint distribution analysis with *t*‐scores and LS presenting larger and more widespread negative ICC clusters outside of auditory brain regions. Echo‐combined LS demonstrated larger between‐session differences in conjunction with increased between‐participant variance, which may explain the non‐significant results when directly comparing intra‐voxel reliability between LS and EPI. Additionally, intra‐voxel reliability was assessed in an ROI of auditory brain regions where both sequences showed high ICC values, potentially minimising between‐sequence differences.

Despite a direct comparison of clear speech with and without mixed EPI‐like scanner noise, we were unable to detect any significant differences in either single‐echo or echo‐combined LS which runs contrary to previously reported results for example by Yakunina et al. (2015) or Pellegrino et al. (2022). One possible explanation is limited statistical power, given our smaller sample size (*N* = 10) compared to these studies, although the repeated measures employed should help to compensate for this lack somewhat and should be counted as a strength of the current study. The absence of significant effects is unlikely to be due to an inadequate or imperceptible acoustic manipulation as we carefully matched the SNR of the mixed condition to realistic scanner environments by directly recording the background noise of our EPI sequence and by accounting for the muffling effects of our ear defenders (approximately −20 dB). These steps made sure of a close approximation of in‐scanner listening conditions. It is possible that the simulated scanner noise, whereas perceptible, did not place sufficient demands on the brain regions implicated in previous studies to elicit robust BOLD signal increases. Responses to scanner noise may also involve network‐level effects (e.g., connectivity changes) which were not captured by our BOLD signal analysis but could be picked up in the studies cited above. In future studies involving LS, larger sample sizes and different analytic or acquisition approaches (i.e., connectivity analyses) may address these limitations and yield results in line with previous findings.

It is also important to mention that LS inherently is a multi‐echo acquisition technique and offers the associated benefits such as the possibility of echo combination and increased sensitivity to the BOLD signal (Lynch et al. 2020). The relatively long TR (3.48 s) used in this study was chosen to match the EPI sequence to the LS sequence, which at the time of study conception was limited in its temporal resolution, as well as by the dependency between TR, FOV and echo sampling in LS. This design, while reducing the temporal sampling rate of EPI, ensured comparable acquisition timing and spatial parameters. Although current implementations of LS feature relatively long repetition times (> 2.2 s), ongoing sequence development is actively addressing this limitation and will do so in the future, as LS can currently still be considered to be in the early stages of its development. Future implementations of LS are expected to enable substantially shorter TRs, improving temporal resolution. Advanced denoising techniques—such as those implemented in the tedana Python library (Ahmed et al. 2023)—could further improve the SNR of multi‐echo LS data via principal and independent component analysis (Steel et al. 2022). In this study, we chose not to follow such approaches to maintain comparability to EPI, but they represent promising avenues for future research using LS. Methodological decisions such as our approach to echo combination and the comparison to single‐echo EPI align with prior validations of LS fMRI (Damestani et al. 2019, 2021; Dionisio‐Parra et al. 2020; Wiesinger et al. 2019). Furthermore, a comparison to multi‐echo EPI was not deemed appropriate due to the inability of such a sequence to acquire a FID image and technical limitations, namely the inability to adjust echo times to be comparable to LS, making it incompatible with our implementation of multi‐echo LS. A direct comparison between matched multi‐echo EPI and multi‐echo LS would be desirable for future studies to comprehensively rule out that echo‐combined LS results are driven by sequence differences present in this study, preferably once LS is able to reach peak TRs of < 1 s. Another alternative to multi‐echo EPI, which might allow for more time to evaluate additional TEs and which might be considered by future research, is simultaneous multislice (SMS) imaging (Maudsley 1980; Müller 1988), a technique which allows the capture of data from multiple slices of the brain simultaneously. A combined approach of multi‐echo and SMS imaging might also be considered (Boyacioğlu et al. 2015; Olafsson et al. 2015), with the added benefits of increased sensitivity, statistical power, as well as specificity.

In conclusion, we demonstrated that LS fMRI enables high‐quality imaging of speech processing without the confound of scanner noise. Echo‐combined LS showed increased BOLD sensitivity to degraded speech, uncovering engagement in key regions such as the IFG and the precentral gyrus. This underscores the potential of LS as a valuable tool for auditory neuroscience, especially as its development continues. Its silent properties may benefit auditory neuroscience and the research of noise‐sensitive populations such as children and people with autism (Williams et al. 2021).

## Funding

This work was supported by Schweizerischer Nationalfonds zur Förderung der Wissenschaftlichen Forschung (100014_204299).

## Data Availability

The data that support the findings of this study are available from the corresponding author upon reasonable request.
